# Country differences in the link between gender-role attitudes and marital centrality: Evidence from 24 countries

**DOI:** 10.1177/0020715220985922

**Published:** 2021-01-07

**Authors:** Kamila Kolpashnikova, Muzhi Zhou, Man-Yee Kan

**Affiliations:** Department of Sociology, University of Oxford, UK

**Keywords:** Gender revolution, gender-role attitudes, marriage centrality, second demographic transition

## Abstract

This study investigates factors that could explain why the association between the egalitarian gender-role attitudes and the attitudes toward the importance of marriage (marital centrality) differs across societies. Using data from the International Social Survey Programme for 24 countries in 2002 and 2012 and multilevel modeling, we explore whether the Gender Revolution and the Second Demographic Transition frameworks could explain the country-level differences in the association between gender-role attitudes and marital centrality. We find that the negative association between the egalitarian gender-role attitudes and marital centrality is stronger in countries with a higher gender equality level and a higher fertility level. This work highlights the importance of considering the progress of the gender revolution and the second demographic transition to understand the relationship between gender equality and family formation.

## Introduction

The forms of family are diversifying in many industrialized societies. From the 1960s to the 1990s, marriage was on the decline as the number of cohabitation surged, and divorce rates rose (Amato and Rogers, 1997; Glenn, 1991; Wilcox and Nock, 2006). Scholars argue that changes in family life and family forms reflect improved gender equality, where more women participate in the labor market and achieve their career ambitions. To a large extent, more egalitarian gender values, where both women and men contribute to family income and share domestic work, are replacing the older traditional gender ideology over time (Amato and Booth, 1995; Esping-Andersen and Billari, 2015). In terms of family values, public opinions in many western industrialized countries are also shifting to be more supportive of the deinstitutionalization of marriage (Gubernskaya, 2010; Treas et al., 2014).

The aforementioned trends in gender norms and family values over time indicate that the relationship between gender equality and the importance of marriage is negative. The Gender revolution (GR) framework posits that more egalitarian gender attitudes bring about more marital discord and less satisfaction with family life. For instance, in regions suffering from the lack of gender equality in the domestic sphere (e.g., Southern European countries), people with more egalitarian gender attitudes are less likely to marry or have children and likely to divorce (Rindfuss et al., 2016). This argument echoes the Second Demographic Transition (SDT) framework, where the conflict between values relating to marriage and family life and individualistic values are the reasons behind the initial drop in both fertility level and marriage rate at the early stage of SDT (*early-SDT*) (Lesthaeghe and Meekers, 1987; Van De Kaa, 1987, 2002).

Nonetheless, as the GR and the SDT continue, an unexpected increase in fertility level has been observed in countries that are pioneers in gender equality (Rindfuss et al., 2016). The GR framework outlined that when the conflict between the gender roles in both public and private spheres is resolved, the gender revolution has entered the second stage, and we would observe a return of the family, represented by increased fertility level (Esping-Andersen and Billari, 2015). For example, in the Scandinavian region, the normalization of egalitarian beliefs and the erosion of the man-breadwinner/woman-homemaker modalities could be why the total fertility rates are relatively high (Esping-Andersen and Billari, 2015; Goldscheider et al., 2015; McDonald, 2000). The second stage of the gender revolution corresponds to the *mid-SDT* phase, and a relatively high level of fertility is maintained in societies with a high level of gender equality.

So far, we have observed the return of fertility level as the GR and the SDT progress. Has there been a return of marriage as well? Abundant empirical research shows that the relationship between gender equality and family formation is dynamic, which evolves with the progress of the gender revolution (Jalovaara et al., 2017; Pessin, 2018; Zhou and Kan, 2019). Yet, analyses and empirical studies focusing on the subjective relationship between gender equality and marriage are rare and tend to focus on a single society (Katsurada and Sugihara, 2002; Ohlsson Wijk et al., 2018). A broader framework that encompasses more countries across regions beyond Anglo-Saxon and Western European contexts, where countries are at vastly different stages of the GR and the SDT, has not yet been tested.

This article contributes to the literature by providing one of the first global comparisons with countries at different stages of the GR/SDT to gauge a complete and more accurate picture of the relationship between gender attitudes and marital centrality (the importance of marriage relative to other family forms). In this article, we include countries from East Asia, Eastern Europe, Central Europe, Western Europe, North America, and Scandinavia to demonstrate how the link between gender attitudes and marriage centrality differs by the stage of the gender revolution and the second demographic transition. As more countries make progress in the gender revolution and the second demographic transition, this work will provide important predictions for the future of family.

Using the repeated cross-sectional ISSP data for 24 countries in 2002 and 2012 and the United Nations human development and demographic database, our study offers new evidence and explanations for the cross-country associations between egalitarian gender attitudes (EGA) and attitudes toward marriage. The research questions of this study are the following: (1) whether higher levels of EGA contribute to the lower support for marriage centrality, (2) whether there are differences in this relationship across countries, and (3) whether the country disparities in this association are consistent with the GR (increased gender egalitarianism on the societal level) and SDT (increased total fertility rates (TRF)) explanations.

## Gender egalitarian ideology and attitudes toward marriage

For most of history, the institution of marriage was a place to reinforce unequal gender roles (Berk, 1985; Cherlin, 1978). Traditional societal expectations dictate that men need to work, and women do unpaid domestic work, thus, reinforcing spousal specialization in marriage. Centuries of such gender traditionalism and specialization shaped a persistent gender wage and housework performance gap, which we observe now (Cha and Weeden, 2014; England, 2005; Gupta, 2014). Gender attitudes also reverted to more traditional after marriage, particularly among mothers who quit their jobs for the sake of taking care of their families (Baxter et al., 2014; Zhou, 2017).

The past 50 years witnessed a revolution in gender relations with a decline in marriage rates, the prevalence of cohabitation, and the surge of divorce rates in almost all industrialized societies (Esping-Andersen and Billari, 2015; Treas et al., 2014). Nowadays, more women receive higher education and have more opportunities in the labor market than before. For women, marriage, an institution for traditional gender-role display, has become less desirable than self-realization in a career (Becker and Tomes, 1994; Nemoto, 2008). Traditional gender relations also took a toll on egalitarian men. Traditional relations within marriage impose heavy demands on men’s earning capability and prevent those with low socioeconomic status from finding a partner, having offspring, and being more involved in family life (Kalmijn, 2011; Mensch et al., 2005).

Moreover, the work-family conflict is exceptionally high for dual-earner couples in societies where the institutional support for childcare is limited, such as the United States and the United Kingdom. The traditional family expectations lay a higher burden on both women and men who adopted the egalitarian attitudes (Hagqvist et al., 2017). Many working parents, especially mothers, find it challenging to meet the rising expectations of childcare and professional career at the same time. These expectations create a conflict between gender roles within marriage and gender-egalitarian values (Dominguez-Folgueras et al., 2018; Zhou, 2017).

In summary, at the individual level, people who hold more egalitarian gender attitudes are more likely to be unsatisfied with the institution of marriage that emphasizes unequal gender roles and, thus, are less likely to support the importance of marriage.

## Gender attitudes and marital centrality: country/regional differences

The negative effect of egalitarian gender-role attitudes on marital centrality may vary based on the specific social context. In countries where childrearing outside of marriage remains stigmatized, the institution of marriage remains highly valued across the population, even among egalitarian individuals. For instance, in countries like Japan, alternatives to marriage such as extra-marital births lead mostly to social stigma (Hertog, 2009), and egalitarian gender-role attitudes are associated with higher marital satisfaction among Japanese men, but not women (Taniguchi and Kaufman, 2014).

In those societies, the gender inequality level is usually high because there are few alternatives to marriage if one wants to form a family. Married women and men are confined to the traditional gender-role settings. Marriage remains highly attractive to all men because they could do very little around the house. For women, they have few alternatives beyond getting married and having children; otherwise, they need to remain single and childless. The rigidity of the institution of marriage also means that social policies and public opinions leave women with little support to pursue personal goals outside the family. This mismatch between gender roles outside and within the family is viewed as a key reason for the very low fertility levels in countries where traditional family forms remain highly valued, such as Italy and Japan (McDonald, 2000). These countries entered the gender revolution and the second demographic transition but remained at a very early stage (early-GR/SDT), featuring high levels of gender inequality at home together with low fertility rates.

Specifically, in countries at this early stage, the extent of the gender revolution has not reached the level on which it could jeopardize the critical social function of marriage. In other words, the gender revolution has not entered the private sphere. Second, society has not cultivated sufficient individualistic values to offset the strong influence of social norms that emphasize marriage’s centrality. These individualist values are critical to progress to the next stage of the gender revolution and the second demographic transition. People who uphold gender-egalitarian views remain confined by social norms and could still be reluctant to acknowledge other family forms. The relationship between egalitarian gender-role attitudes and marital centrality is expected to be moderate in these societies at the early-GR/SDT stage.

With the deepening of the gender revolution and the second demographic transition, traditional marriage values become incompatible with pursuing personal goals. An important phenomenon is that alternative forms of family emerge and challenge the centrality of “traditional” marriage. Such “new” ways of family arrangement become more accepting of more egalitarian labor division between women and men. Consequently, improved gender equality is then connected to higher fertility rates, driven by childrearing outside of marriage. Based on the gender revolution and the second demographic transition explanations, the gradual diffusion of individualistic values both within and outside of marriage and family engendered these demographic trends and the reversal of fertility level (Lesthaeghe, 2010; Lesthaeghe and Van De Kaa, 1986). Countries with a relatively high level of gender equality and a high level of total fertility rate start to enter the mid-GR/SDT stage.

Specifically, in countries where the gender equality level and the total fertility rate are high, individuals have gained extensive opportunities to fulfill their personal goals both within and outside the institution of marriage through participating in the labor market and having children outside marriage. Those who hold more egalitarian gender views have tremendous power to devalue the importance or centrality of marriage. Thus, in these mid-GR/SDT countries, egalitarian women and men would show higher disapproval toward marital centrality.

## Hypotheses

Based on the earlier discussion, we propose three hypotheses:

- *Hypothesis 1.* People with more gender-egalitarian attitudes are less supportive of traditional marital centrality.
- *Hypothesis 2.* The negative link between gender egalitarianism and marriage centrality is stronger in more gender-egalitarian countries (mid-GR/SDT) and weaker in less gender-egalitarian countries (early-GR/SDT).
- *Hypothesis 3.* The negative link between gender egalitarianism and marriage centrality is stronger in countries with higher fertility rates (mid-GR/SDT) and weaker in countries with lower TFR (early-GR/SDT).

## Data and method

### Data and sample

To test the link between gender attitudes and marriage centrality across countries, we use data from the International Social Survey Programme’s (ISSP) Family and Changing Gender Roles modules in 2002 and 2012 (ISSP Research Group, 2016). We chose the Family module because it has consistent marital centrality and egalitarian gender attitudes measures. ISSP interviewed individuals above 18 years old using various interview collection strategies: face-to-face interviews with a standardized questionnaire, paper-and-pencil postal surveys, and web surveys.

We select 24 countries that had all variables of interest in both survey periods. For the data visualization purpose, we classify those countries by social-welfare regime (Esping-Andersen, 1990), levels of socioeconomic development, and geographical locations, anticipating that these factors will indicate the progress of the SDT and the gender revolution. Following the categorization in Esping-Andersen (1990), we, first, identify (1) liberal regime states—Australia, the United Kingdom, and the United States, where the institutional support for families are expected to be limited or based on family income and means; (2) socio-democratic regime states—Denmark, Finland, Norway, Sweden, where there is strong institutional support to improve gender equality, and the overall level of gender equality is the highest; and (3) conservative welfare states—Austria, Belgium, France, Germany, the Netherlands, Switzerland, where the institutional support is modest and often family-based. Following a supporting work of Ferrera (1996), we also add the (4) Mediterranean welfare states—Spain, Portugal, where traditional gender relations in marriage and family ties are highly valued (Billari and Kohler, 2004). Furthermore, we group the Eastern European block based on their geographical commonalities and Soviet history. They are (5) Eastern European states—Czech Republic, Hungary, Latvia, Poland, Russia, Slovenia, Slovakia, which experienced the collapse of the Soviet Union and where individualist ideas are less encouraged and valued compared to the Western European countries (Bakacsi et al., 2002). The East Asian countries stand apart from the (mostly) European countries above, but both share common history and culture because Japan occupied Taiwan in the 18th–19th centuries, so we also classify (6) East Asian regimes—Japan and Taiwan, where the Confucian family values that emphasize women’s subordinate position prevail and individualist values rarely overcome the collectivist goals (Hamamura, 2011), although recent research shows that in East Asian societies the changes are happening, as well (Yang and Yen, 2011).

We further restricted the sample to those between 20 and 59 years of age to analyze the active labor force participants. After dropping observations with missing values on any variable of interest, the final analytical sample contained 39,253 individuals—18,109 in 2002 and 21,144 in 2012. In total, 44% were males, and 56% were females. Please refer to Supplemental Appendix Tables A1, A2 (a, b), and A3 (a, b) for details on the sample distributions.

**Table 1.** Predicting marital centrality and test gender differences, ISSP 2002 & 2012.

	Baseline 2002	EGA × Gender 2002	Baseline 2012	EGA × Gender 2012
Standardized EGA	−0.247*** (0.015)	−0.247*** (0.016)	−0.287*** (0.016)	−0.285*** (0.017)
Men	0.094*** (0.013)	0.094*** (0.013)	0.104*** (0.012)	0.105*** (0.012)
Men # Standardized EGA		−0.000 (0.012)		−0.004 (0.012)
Partnered (ref: Single)	0.332*** (0.018)	0.332*** (0.018)	0.337*** (0.016)	0.337*** (0.016)
Sep/Div/Wid	0.047* (0.024)	0.047* (0.024)	0.008 (0.022)	0.008 (0.022)
One child (ref: No child)	−0.053*** (0.016)	−0.053*** (0.016)	−0.043** (0.016)	−0.043** (0.016)
Two children	−0.036 (0.019)	−0.036 (0.019)	−0.043* (0.018)	−0.043* (0.018)
Three children	0.053 (0.031)	0.053 (0.031)	0.057* (0.025)	0.057* (0.025)
Part-time working (ref: Full-time working)	−0.016 (0.020)	−0.016 (0.020)	−0.050** (0.016)	−0.050** (0.016)
Not working	0.012 (0.015)	0.012 (0.015)	0.016 (0.015)	0.016 (0.015)
Secondary (ref: < Sec education)	0.010 (0.016)	0.010 (0.016)	0.039* (0.017)	0.039* (0.017)
>Second education	0.092*** (0.016)	0.092*** (0.016)	0.059*** (0.016)	0.059*** (0.016)
Has religion	0.226*** (0.015)	0.226*** (0.015)	0.274*** (0.013)	0.274*** (0.013)
Standardized age	0.132*** (0.008)	0.132*** (0.008)	0.051*** (0.007)	0.051*** (0.007)
Standardized age squared	0.058*** (0.007)	0.058*** (0.007)	0.053*** (0.007)	0.053*** (0.007)
Intercept	−0.442*** (0.082)	−0.442*** (0.082)	−0.483*** (0.079)	−0.483*** (0.079)
SD (EGA)	0.067*** (0.012)	0.067*** (0.012)	0.072*** (0.012)	0.072*** (0.012)
SD (Intercept)	0.385*** (0.056)	0.385*** (0.056)	0.369*** (0.054)	0.369*** (0.054)
Correlation (EGA, Intercept)	0.327 (0.207)	0.327 (0.207)	0.689*** (0.130)	0.689*** (0.130)
SD (Residual)	0.799*** (0.004)	0.799*** (0.004)	0.815*** (0.004)	0.815*** (0.004)
Observations	18,109	18,109	21,144	21,144

EGA: egalitarian gender attitudes.

Standard errors in parentheses.

* *p* < 0.05; **p < 0.01; ***p < 0.001.

### Measures and analytical strategy

The dependent variable is marital centrality. This variable is measured by a scale of views toward the importance of marriage against other family forms. A higher score indicates that people are more supportive of the institution of marriage (research using the same measures includes Gubernskaya, 2010; Treas et al., 2014).

#### Attitudes toward marital centrality

In both years, there are five items: (1) “Married people are generally happier than unmarried people,” (2) “People who want children ought to get married,” (3) “One parent can bring up a child as well as two parents together,” (4) “It is all right for a couple to live together without intending to get married,” and (5) “Divorce is usually the best solution when a couple cannot seem to work out their marriage problems.” The response options range from “1 = strongly agree” to “5 = strongly disagree.” We recode the Spanish attitudes response option in the ISSP 2012 “cannot choose” as “neither agree nor disagree.” The questions (1) and (2) were reverse-coded. After using principal factor analysis to examine whether all items measure the same attitudes. Three items (items 1, 2, and 4) are selected and summed up. This variable ranges between 3 and 15, with a mean value of 7.83 and a standard deviation of 2.82. Cronbach’s alpha on the scale was 0.675, indicating a relatively high level of internal reliability. The internal reliability increased from 0.648 in 2002 to 0.696 in 2012. We standardize this variable to have a mean value of zero and a standard deviation of 1. Items (1), (2), and (4) reflect people’s views about the role of marriage in determining happiness, childrearing, and sharing lives. The higher the value is, the higher the support for marital centrality.

#### Egalitarian gender-role attitudes (EGA)

The key predictor is an individual’s attitudes toward traditional gender roles. Seven items are asked consistently in both years: (1) “Warm relationship with children as a not working mom,” (2) “Preschool child is likely to suffer if mom works,” (3) “Family life suffers when the woman has full-time job,” (4) “What women really want is home and kids,” (5) “Being a housewife is as fulfilling as working for pay,” (6) “Both should contribute to household income,” (7) “Men’s job earn money, women’s job look after the home.” The response options range from “1 = strongly agree” to “5 = strongly disagree.” Like the traditional marital attitudes scale, we recode responses in the Spanish data “cannot choose” as “neither agree nor disagree.” We reverse code items (1) and (6) so that a higher score represents stronger support for women’s employment.

Principal factor analysis showed that items 1, 2, 3, 4, 5, and 7 were consistently measuring people’s gender-role attitudes. We sum up these six items. The resulting values range between 6 and 30, with a mean of 19.93 and a standard deviation of 4.85. The Cronbach’s alpha for the scale was 0.739 for the full sample, 0.712 for the 2002 sample, and 0.727 for the 2012 sample. We standardize this variable (z-scores) to have a mean value of zero and a standard deviation of 1. The higher values indicate higher support of gender-equal roles at home and at work. We, therefore, name this variable as egalitarian gender attitudes. These statements are widely used in other nationally representative surveys to measure gender-role attitudes (Davis and Greenstein, 2009). Selecting these variables should make this work highly comparable with previous studies that studied gender-role attitudes.

#### Country-level variables

We use two country-level indicators to investigate whether the effect of the EGA on marital centrality varies based on these indicators. First, we include the Gender Inequality Index (GII) from the United Nations dataset. This index measures,

“gender inequalities in three important aspects of human development—reproductive health, measured by maternal mortality ratio and adolescent birth rates; empowerment, measured by the proportion of parliamentary seats occupied by females and proportion of adult females and males aged 25 years and older with at least some secondary education; and economic status, expressed as labor market participation and measured by labor force participation rate of female and male populations aged 15 years and older” (United Nations Development Programme, 2019).

The higher the value of GII is, the less gender-equal the country is. In our sample, this value has a mean of 0.146 and a standard deviation of 0.08 across all countries.

The second country-level indicator is the TFR, downloaded from the United Nations World Fertility Data (United Nations, 2019), measuring the average number of children that a woman would have over her lifetime. This value has a mean of 1.56 and a standard deviation of 0.25 across all 24 countries in the country sample.

For detailed information on country-level indicators, please refer to Supplemental Appendix Table A4.

#### Other variables

We further add several sociodemographic controls, which are correlated with both gender-role attitudes and attitudes toward marriage, following others’ work (Gubernskaya, 2010; Kalmijn, 2013; Treas et al., 2014). Gender is for two modal genders (0 = “Women” (ref.), 1 = “Men”). We control for marital status (“1 = single (ref.),” “2 = married/cohabiting,” and “3 = separated/divorced/widowed”). Marital status questions are not asked consistently across countries and over the years. In 2002, cohabitation status is not considered. In 2012, in the countries where cohabitation and marriage were asked (e.g. France, Belgium, Norway), cohabitation was mostly legally and socially not distinguishable from marriage. In more traditional societies (e.g. Japan, Poland, Portugal), the original question only asked whether the respondent was married. Employment status is a categorical variable (1 = “employed full-time” (ref.), 2 = “employed part-time,” 3 = “not employed”). Education is also a categorical variable (1 = “less than secondary education” (ref.), 2 = “secondary education,” 3 = “above secondary education”). We also control for being an atheist (1 = “atheist,” 0 = “has a religion”). The models also include the number of children below the age of 18 (0 = “no children” (ref.), 1 = “1 child,” 2 = “2 children,” and “3 = “more than 2 children”). The age variable is standardized, and the squared term was also added.

### Models

We run ordinary least-squares (OLS) for each country first and then multilevel random intercept and random slope (mixed-effects) models pooling all countries in our analysis (STATA 16.1 code: *mixed*). We have a two-level data structure with individuals nested in countries. The baseline model for the multilevel analysis is the following (separately for each year)

$$MC_{ij}=\alpha_{0}+\gamma EGA_{ij}+\beta_{1j}GII_{j}+\beta_{2j}TFR_{j}+\beta_{3j}X_{ij}+u_{0j}+u_{1j}EGA_{ij}+\varepsilon_{ij}$$

where $MC_{ij}$ is the level of marriage centrality for an individual *i* in country *j* (*j* = 1, . . ., 24), γ—fixed effects of the EGA, $\beta_{1j}$ to $\beta_{2j}$—fixed effects of country-level indicators ($GII_{j}$, $TFR_{j}$), $\beta_{3j}$—fixed effects of control variables $X_{ij}$, $u_{0j}$—random effects with a standard deviation SD (Intercept), $u_{1j}$—random effects of the EGA with a standard deviation SD (EGA), and $\varepsilon_{ij}$—Residual. $u_{0j}$ and $u_{1j}$ are allowed to be correlated, and this correlation is significant with *p* < 0.01 by likelihood-ratio test.

## Results

### Descriptive results

**Figure 1.**
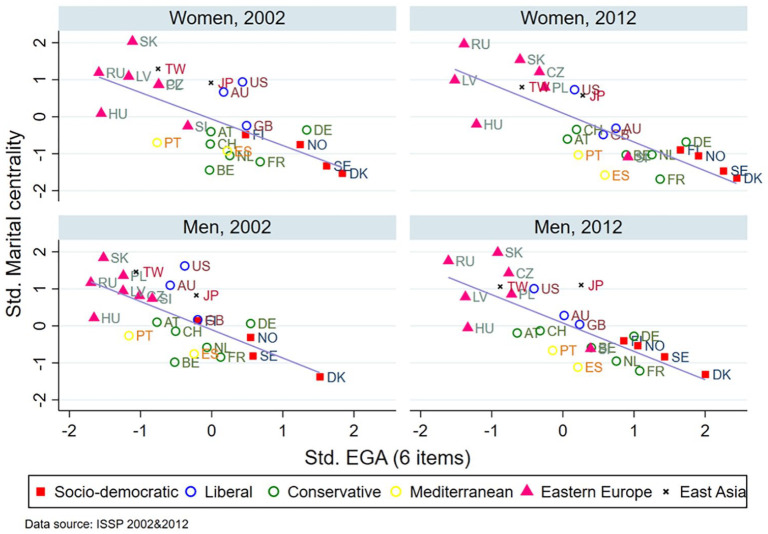
Mean values of the EGA and marital centrality by gender, year, and country.

Figure 1 plots the mean values of the standardized EGA and the standardized marital centrality scores by gender and year for each country (based on Supplemental Appendix Table A4). We fit a linear regression line across those countries. We colored the countries broadly based on their geographical location or cultural or welfare similarities as discussed earlier. At the country-level, the relationship between the EGA and marital centrality is negative. The higher the value of the EGA is in a certain country, the lower the level of marital centrality is. Nordic countries and those in Eastern European countries lie at the opposite ends of these distributions.

**Figure 2.**
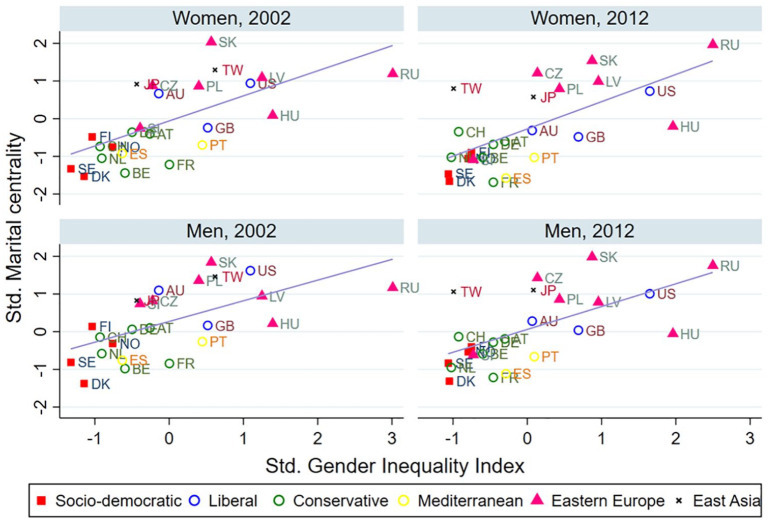
Mean values of GII and marital centrality by gender, year, and country.

**Figure 3.**
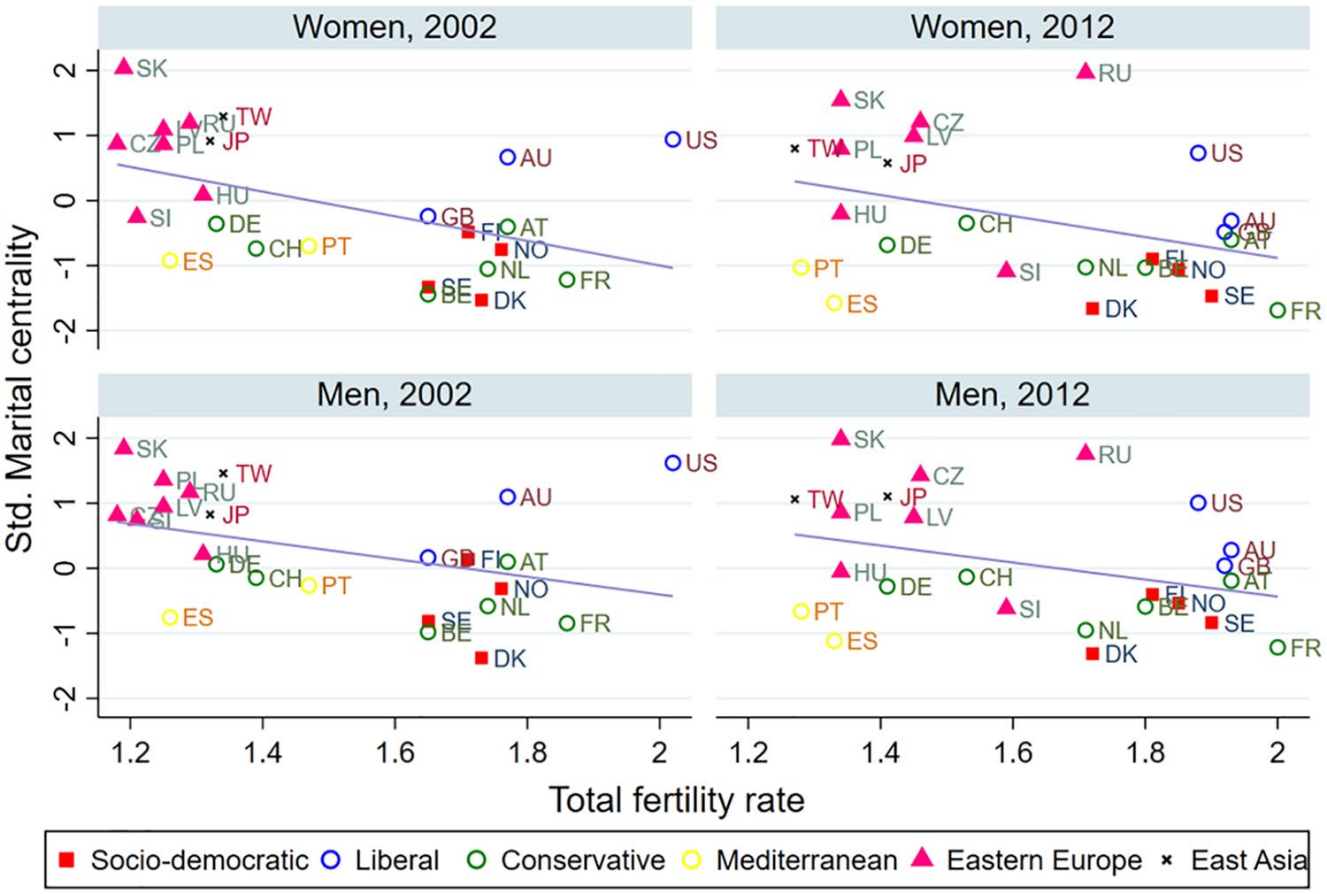
Mean values of TFR and marital centrality by gender, year, and country.

Figures 2 and 3 present the two country-level indicators (GII and TFR) and their relationships with the country-level measures of marital centrality by sex. First, the higher the level of gender inequality is in a country, the higher the level of martial centrality is (Figure 2). This finding indicates that countries with higher levels of gender inequality are more likely to have higher support for traditional attitudes toward marriage. Second, the higher the TFR, the lower the martial centrality (Figure 3), which is mainly driven by the low fertility levels in the Eastern European and East Asian countries. This finding suggests that the reversal in fertility rates is expected when and if the progression of gender egalitarianism and change in traditional marriage attitudes are also present, as some of the previous literature showed (Esping-Andersen and Billari, 2015).

### OLS regression analysis

**Figure 4.**
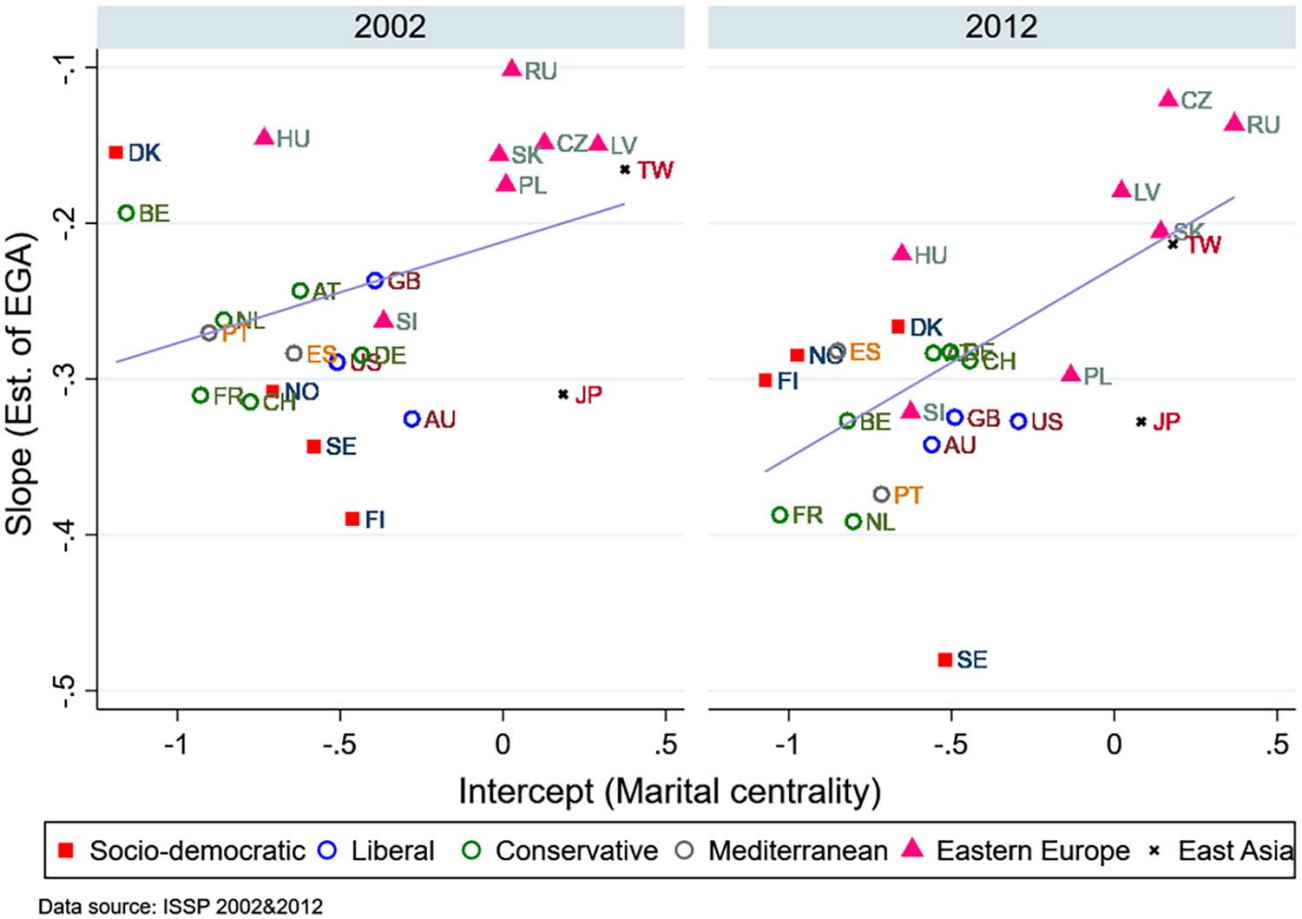
The estimated coefficient of the EGA in each country.

The above results were based on country-level bivariate analyses. To test factors that moderate the effect of the individual-level EGA, we run identical OLS regressions predicting marital centrality using the individuals’ EGA and control variables in each country and plot the estimated coefficients of the EGA in Figure 4.

First, the estimated effects of the EGA are negative in all countries. Second, the EGA estimates vary substantially across countries, from approximately −0.10 in Russia to −0.38 in Finland in 2002 and −0.13 in Czech to −0.47 in Sweden in 2012. From 2002 to 2012, the negative association became stronger, for example, in France, Belgium, and Netherland. Finally, in countries where the intercept values were higher, the estimated values of the EGA were closer to zero. This means that the EGA’s negative effect on marital centrality is smaller in countries where the overall support for the institution of marriage remains high (early-GR/SDT stage). Conversely, in countries where the support for marital centrality is lower (mid-GR/SDT stage), the relationship between the EGA and marital centrality is stronger (more negative). Overall, we find support for Hypothesis 1 in all countries using the OLS regression.

After we confirm the existence of heterogeneity in the effects of the EGA on martial centrality, we would like to know which country-level indicators could explain this considerable variation of the effect of the EGA across countries.

### Multilevel regression results

We pool all countries together and use random intercept random-slope multilevel models. Both the intercept and the estimated effect of the EGA (or the slope) are also allowed to vary across countries.

We first test whether there is a gender difference in the EGA’s effect on marital centrality in each survey year. These models do not include the country-level measures. The results are presented in Table 1.

The negative effect of the EGA is −0.247 in 2002 and −0.287 in 2012. A supplementary analysis pooling the 2 years using the interaction term between the EGA and the year variables shows that the estimate is statistically significantly more negative in 2012 than in 2002 (*p* = 0.001). Thus, the negative effect of the EGA on marital centrality becomes stronger in the later period. The results also confirm the positive correlation between the EGA estimates and the intercept, which changes from 0.327 in 2002 to 0.689 in 2012. This result confirms that the EGA’s negative effect on marital centrality is weaker in countries with a high level of marital centrality.

Our models also show that men, people in a partnership, those who have a religion hold stronger beliefs in marital centrality. The estimate of the educational variables changes between 2002 and 2012, but generally, the more educated people, especially those with an above secondary level of education, including college and university education, have higher marital centrality levels. This finding is consistent with previous literature, which notes that marriage has become a prestige indicator for more socio-economic power in countries with more equal gender-roles or a high- income inequality (Cherlin, 2010; Kalmijn, 2013). We do not find that the EGA’s effect differs between women and men through the interaction term. Therefore, in the following analyses, we pool samples of women and men together.

In Table 2, we test whether the selected country-level indicators could explain the variation in the EGA estimates across countries in 2002 and 2012. We now include the country-level indicators (GII and TFR) and their interactions with the EGA. The new models help examine whether those country-level indicators would explain the variance of the EGA estimates across countries.

**Table 2.** Predicting marital centrality with country-level indicators, key variables.

	Model 1	Model 2	Model 3	Model 4	Model 1	Model 2	Model 3	Model 4
	Baseline 2002	EGA × GII 2002	EGA × TFR 2002	EGA × GII and EGA × TFR 2002	Baseline 2012	EGA × GII 2012	EGA × TFR 2012	EGA × GII and EGA × TFR 2012
Standardized EGA	−0.247*** (0.015)	−0.333*** (0.030)	−0.030 (0.081)	−0.151 (0.084)	−0.287*** (0.016)	−0.366*** (0.028)	−0.071 (0.098)	−0.181* (0.090)
Gender inequality index (GII)	2.862*** (0.759)	2.346** (0.769)	2.836*** (0.761)	2.442** (0.768)	1.831* (0.801)	3.137*** (0.890)	1.815* (0.800)	3.016*** (0.878)
Total fertility rate (TFR)	−0.522* (0.249)	−0.524* (0.247)	−0.372 (0.255)	−0.409 (0.252)	−0.013 (0.228)	−0.011 (0.227)	−0.280 (0.258)	−0.230 (0.249)
Standardized EGA × GII		0.496** (0.159)		0.404** (0.148)		0.643** (0.196)		0.585** (0.181)
Standardized EGA × TFR			−0.143** (0.053)	−0.109* (0.048)			−0.133* (0.060)	−0.109* (0.051)
Intercept	−0.148 (0.434)	−0.055 (0.432)	−0.368 (0.442)	−0.243 (0.440)	−0.688 (0.402)	−0.850* (0.402)	−0.252 (0.447)	−0.479 (0.437)
SD (EGA)	0.067*** (0.012)	0.054*** (0.010)	0.055*** (0.011)	0.047*** (0.010)	0.072*** (0.012)	0.057*** (0.011)	0.064*** (0.012)	0.051*** (0.010)
SD (Intercept)	0.299*** (0.045)	0.294*** (0.043)	0.296*** (0.044)	0.293*** (0.043)	0.312*** (0.051)	0.298*** (0.045)	0.305*** (0.048)	0.293*** (0.043)
Correlation (EGA, Intercept)	−0.299 (0.275)	−0.269 (0.249)	−0.259 (0.260)	−0.243 (0.241)	0.559* (0.206)	0.510* (0.201)	0.536* (0.204)	0.496* (0.197)
SD (Residual)	0.799*** (0.004)	0.799*** (0.004)	0.799*** (0.004)	0.799*** (0.004)	0.815*** (0.004)	0.815*** (0.004)	0.815*** (0.004)	0.815*** (0.004)
Observations	18,109	18,109	18,109	18,109	21,144	21,144	21,144	21,144

EGA: egalitarian gender attitudes; GII: gender inequality index; TFR: total fertility rates.

Standard errors in parentheses.

* *p* < 0.05; **p < 0.01; ***p < 0.001.

In the baseline models in 2002 and 2012, the EGA’s effect remains unchanged after the inclusion of country-level indicators, compared with Table 1. In both years, individuals show a higher level of marital centrality in countries with higher values of GII (Model 1). In 2002, the support for marital centrality was lower in countries with higher fertility levels. If we add an interaction term between the individual-level EGA and the country-level GII (2002 Model 2), the interaction term’s estimated coefficient is 0.496 and is statistically significant. The interaction term’s estimate in 2012 is even larger, with a value of 0.643 (2012 Model 2). Thus, the negative effect of the EGA is weaker among individuals in countries with higher levels of gender inequality. This means that egalitarian women and men living in more gender-equal countries oppose the importance of marriage more strongly. These findings support Hypothesis 2. Adding the interaction term between the EGA and the country-level GII could explain $35\%\left(1-\frac{0.054^{2}}{0.067^{2}}\right)$ and $37\%\left(1-\frac{0.057^{2}}{0.072^{2}}\right)$ of the total variance in the effect of the EGA across countries in 2002 and 2012, respectively, which is a substantial amount. Therefore, the inclusion of the EGA and GII interaction could explain a considerable proportion of the variance in the EGA effect across countries.

To test Hypothesis 3, from the baseline model, we add the interaction between the EGA and the TFR. In both years, the interaction term’s estimate shows that the EGA’s negative effect is more substantial when the country-level TFR is higher. The reduction in the proportion of variance of the EGA effect is similar to the model with the interaction between the EGA and the GII (Model 2). This finding supports Hypothesis 3.

We now add both the interactions between the EGA and the GII and the EGA and the TFR in Model 4. Both estimates of these two interaction terms are statistically significant in both years, and their estimates remain mainly changed. Adding the two interaction terms together could explain $49\%\left(1-\frac{0.047^{2}}{0.067^{2}}\right)$ and $50\%\left(1-\frac{0.051^{2}}{0.072^{2}}\right)$ of the total variance in the effect of the EGA in 2002 and 2012, respectively. Therefore, the two country-level indicators jointly provide powerful explanations of EGA’s different effects across countries and lend strong support to both the GR and SDT theories.

We have also considered two other country-level indicators, which are the proportion of people ever married (the selected age group was 45–49 years) and the proportion of people who reported to have a religion in each country of the sample, to test whether the moderating roles of GII and TFR still hold. These variables are likely to be correlated with both country-level GII and TFR as well as marital centrality (De Graaf and Kalmijn, 2006; Kalmijn, 2009; Lesthaeghe and Meekers, 1987). Adding these additional country-level variables will not change the results, and these variables, together with their interaction terms with EGA, are not statistically significant at 0.05 level.

In summary, the above results show support for the role of gender inequality on the societal level (Gender Revolution Framework) and the total fertility rates (Second Demographic Transition) in moderating the association between the EGA and marital centrality. EGA’s negative effect on marital centrality is stronger in more gender-equal countries and countries with higher fertility levels. The classic example is that the EGA’s negative effect is much stronger in Nordic countries than in Eastern European countries and Taiwan, which are characterized by higher levels of gender traditionalism and lower fertility rates, as shown in Figures 2 and 3.

### Sensitivity analysis

Given that the number of units at the country-level is small (*n* = 24), we conduct an outlier analysis to test whether specific countries dominate the effects of the EGA and the EGA × GII and the EGA × TFR interaction terms. We use the DFBETA statistics that measure the influence of an individual case (a country in this example) on the estimated effects (STATA code: *mltcooksd*). This test compares a model with and without that case. It is defined as the difference between regression coefficients in the full sample (with all countries) and the sample without a specific country. The difference is then divided by the standard error of the estimate in the sample without the tested country (Belsley et al., 2005). This method is commonly used in multilevel modeling to check how influential cases are in those models (Kalmijn, 2009; Van Der Meer et al., 2010).

In 2002, the DFBETA statistics identified four outlier countries—the United States, Slovakia, Russia, and Denmark. The most influential country was the United States, which had a high GII and a high TFR with relatively high support for marriage centrality. These countries have a strong influence on the estimation of the EGA and the two interaction terms of interest. In 2012, the outlier countries were the Czech Republic, Hungary, Russia, and Taiwan. In Table 3, we estimate the association between the EGA and marital centrality without these strongly influential countries to test whether the results in Model 4 in Table 2 are replicable without the outlier countries.

**Table 3.** Predicting marital centrality (influencing cases excluded), ISSP 2002 and 2012, key variables.

	Interaction 2002	Interaction 2012
Standardized EGA	−0.204* (0.087)	−0.214* (0.087)
Gender inequality index	1.746 (1.044)	4.317*** (0.938)
Total fertility rate	−0.593* (0.279)	−0.105 (0.206)
Standardized EGA × gender inequality index	0.568** (0.189)	0.412 (0.221)
Standardized EGA × total fertility rate	−0.097* (0.048)	−0.085 (0.047)
Intercept	0.150 (0.504)	−0.890* (0.379)
SD (EGA)	0.033*** (0.010)	0.038*** (0.010)
SD (Intercept)	0.261*** (0.042)	0.216*** (0.035)
Correlation (EGA, Intercept)	−0.160 (0.318)	0.148 (0.298)
SD (Residual)	0.786*** (0.005)	0.819*** (0.004)
Observations	14930	16934

EGA: egalitarian gender attitudes.

Standard errors in parentheses.

* *p* < 0.05; **p < 0.01; ***p < 0.001.

After excluding these highly influential countries, the estimates in Tables 3 and 2 remain similar. The EGA’s negative effect remains weaker in countries with a higher GII and stronger in countries with a higher TFR. In conclusion, the interaction effects of the GII and the TFR are not sensitive to the selection of country subsets.

## Discussions and conclusions

This article analyzed the relationship between egalitarian gender attitudes and attitudes toward marriage across 24 countries, covering a wide range of socio-political and social-welfare regimes and geographical locations. We confirm that the association between the egalitarian gender attitudes and supportive attitudes toward marriage is negative across societies. We also demonstrate that this association differs across countries, and the GR and the SDT frameworks could explain these differences. This work contributes to the previous literature on the relationship between gender equality and family formation by providing new evidence that societal gender inequality and total fertility rates play an essential role in the relationship between individual gender attitudes and marital centrality.

This article first demonstrated that the negative association between the egalitarian gender attitudes and marital centrality is stronger in countries that have entered the mid-GR/SDT stage, where gender equality and fertility levels are relatively high. A classic example is those Nordic countries. In these countries, the negative association between individual egalitarianism is stronger than in countries with lower overall gender egalitarianism, such as Eastern European countries. These findings confirm that the gender revolution on the societal level can affect the results at the individual level.

Second, the strength of the association between individual gender egalitarianism and marriage centrality is more robust in countries with higher TRF, which are also countries where the childbearing/childrearing and the institution of marriage have detached. By contrast, both women and men in Eastern European countries are on the other side. In these countries, the level of support for the institution of marriage is less dependent on their level of gender egalitarianism. Given the overall high level of support for the importance of marriage, this finding could be attributed to the legacy of collectivism, which encourages conformism within societies and the reversal to more traditional gender roles following the Soviet Union’s fall. Overall, the deinstitutionalization of marriage in society is critical in determining how powerful one’s individual gender-role attitudes are associated with the attitudes toward marriage. The progress in the second demographic transition matters. A relatively high level of individualist values that are diffused into the private sphere is critical to the association between personal gender-role attitudes and marital centrality.

Finally, the inclusion of selected country-level variables lends strong support to the explanatory power of the GR and the SDT in understanding cross-country differences. GII and TFR jointly explain half of the cross-country variations in the relationship between the EGA and marital centrality. The application of multilevel modeling and the analysis of variables cross levels offer a nice example to demonstrate how theoretical frameworks can be translated into empirical analyses. The GR and SDT frameworks set the societal context that moderates the individual-level relationship between attitudes toward gender roles and marriage. Therefore, it is critical to consider both gender relations and the progress of demographic transition in a specific society to understand the relationship between gender equality and family formation.

One limitation of this work is the different meanings assigned to gender egalitarianism and marriage centrality measures across various counties. It is not always clear how western values might be applied in non-western countries. We noted that the levels of internal reliability for the subjective measures of gender-roles and marriage are higher in Nordic and Western European countries than in East Asian and Eastern European countries. We call for more studies to focus on this cross-cultural issue on attitudinal measures. Moreover, different groups may be at different stages of the gender revolution (e.g. by socioeconomic power, race, and ethnicity). Stanik et al. (2013) reported that the egalitarian division of labor is associated with higher marital satisfaction levels among Black Americans than among Whites. This finding suggests that different groups of people in the same society can be at different stages of the GR and SDT. Future research could explore this avenue.

## Conclusion

This article contributes to the scholarship on the effects of the gender revolution and the Second Demographic Transition on the link between individual gender attitudes and their views toward marriage. It provides one of the first global comparisons to gauge a more comprehensive picture of the relationship between gender attitudes and marital centrality at different stages of GR/SDT. In particular, the results bring to the fore the importance of considering not only gender relations but also the progress of the GR and SDT across countries to understand the relationship between gender equality and family. The findings contribute to the existing theoretical framework on GR and SDT, which conceptualized the ideas but did not test them on the attitudinal dimension across states at the different stages of GR and SDT (Goldscheider et al., 2015; Lesthaeghe, 2010; Lesthaeghe and Van De Kaa, 1986).

Furthermore, our study illustrates how societal levels of gender egalitarianism on par with the total fertility rates can moderate the individual-level association between gender attitudes and marital centrality. The presence of this moderation effect contributes to the theoretical underpinnings of prior family studies research that previously resulted in mixed and fragmented findings across different societies (Hagqvist et al., 2017; Hertog, 2009; McDonald, 2000; Taniguchi and Kaufman, 2014), tying them together with the GR/SDT literature (Goldscheider et al., 2015; Lesthaeghe, 2010; Lesthaeghe and Van De Kaa, 1986). It helps to further our theoretical and empirical understanding of how the societal processes are intertwined with the individual-level processes and illustrate these processes using empirical data, which was not yet done before.

Broadening the analytical scope to incorporate more countries helps elaborate and test existing theories. The combination of countries of different levels of individualism/collectivism and different gender norms, as in this article, provides an excellent scenario to align SDT and GR frameworks. We call for more studies to understand family development when women’s and men’s roles are expected to continue converging in the future. According to this article’s findings, as more and more countries make progress in the gender revolution and the second demographic transition, the negative link between gender-equal attitudes and marital centrality will be enhanced. The deinstitutionalization of marriage will be more common across countries.

## Supplemental Material

sj-pdf-1-cos-10.1177_0020715220985922.pdf – Supplemental material for Country differences in the link between gender-role attitudes and marital centrality: Evidence from 24 countries Click here for additional data file. Supplemental material, sj-pdf-1-cos-10.1177_0020715220985922.pdf for Country differences in the link between gender-role attitudes and marital centrality: Evidence from 24 countries by Kamila Kolpashnikova, Muzhi Zhou and Man-Yee Kan in International Journal of Comparative Sociology

## References

[bibr1-0020715220985922] Amato PR Booth A (1995) Changes in gender role attitudes and perceived marital quality. American Sociological Review 60: 58–66.

[bibr2-0020715220985922] Amato PR Rogers SJ (1997) A longitudinal study of marital problems and subsequent divorce. Journal of Marriage and Family 59: 612–624.

[bibr3-0020715220985922] Bakacsi G Sándor T András K, et al. (2002) Eastern European cluster: Tradition and transition. Journal of World Business 37: 69–80.

[bibr4-0020715220985922] Baxter J Buchler S Perales F, et al. (2014) A life-changing event: First births and men’s and women’s attitudes to mothering and gender divisions of labor. Social Forces 93: 989–1014.

[bibr5-0020715220985922] Belsley DA Kuh E Welsch RE (2005) Regression Diagnostics: Identifying Influential Data and Sources of Collinearity. Hoboken: Wiley.

[bibr6-0020715220985922] Becker GS Tomes N (1994) Human capital and the rise and fall of families. In: Becker GS (ed.) Human Capital: A Theoretical and Empirical Analysis with Special Reference to Education. Chicago, IL The University of Chicago Press, pp. 257–298.

[bibr7-0020715220985922] Berk SF (1985) The Gender Factory: The Apportionment of Work in American Households. New York: Plenum Press.

[bibr8-0020715220985922] Billari F Kohler H-P (2004) Patterns of low and lowest-low fertility in Europe. Population Studies 58: 161–176. 15204251 10.1080/0032472042000213695

[bibr9-0020715220985922] Cha Y Weeden K (2014) Overwork and the slow convergence in the gender gap in wages. American Sociological Review 79: 457–484.

[bibr10-0020715220985922] Cherlin AJ (1978) Remarriage as an incomplete institution. American Journal of Sociology 84: 634–650.

[bibr11-0020715220985922] Cherlin AJ (2010) The Marriage-Go-Round: The State of Marriage and the Family in America Today. New York: Knopf Doubleday Publishing Group.

[bibr12-0020715220985922] Davis SN Greenstein TN (2009) Gender ideology: Components, predictors, and consequences. Annual Review of Sociology 35: 87–105.

[bibr13-0020715220985922] De Graaf PM Kalmijn M (2006) Change and stability in the social determinants of divorce: A comparison of marriage Cohorts in the Netherlands. European Sociological Review 22: 561–572.

[bibr14-0020715220985922] Dominguez-Folgueras M Jurado-Guerrero T Botía-Morillas C (2018) Against the odds? Keeping a nontraditional division of domestic work after first parenthood in Spain. Journal of Family Issues 39: 1855–1879.

[bibr15-0020715220985922] England P (2005) Gender inequality in labor markets: The role of motherhood and segregation. Social Politics 12: 264–288.

[bibr16-0020715220985922] Esping-Andersen G (1990) The Three Worlds of Welfare Capitalism. Cambridge: Polity Press.

[bibr17-0020715220985922] Esping-Andersen G Billari FC (2015) Re-theorizing family demographics. Population and Development Review 41: 1–31.

[bibr18-0020715220985922] Ferrera M (1996) The “southern model” of welfare in social Europe. Journal of European Social Policy 6: 17–37.

[bibr19-0020715220985922] Glenn ND (1991) The recent trend in marital success in the United States. Journal of Marriage and Family 53: 261–270.

[bibr20-0020715220985922] Goldscheider F Bernhardt E Lappegard T (2015) The gender revolution: A framework for understanding changing family and demographic behavior. Population and Development Review 41: 207–239.

[bibr21-0020715220985922] Gubernskaya Z (2010) Changing attitudes toward marriage and children in six countries. Sociological Perspectives 53: 179–200.

[bibr22-0020715220985922] Gupta N (2014) Gender wage gap in the last ten years: A case study of India. ORF Working Paper Series. Available at: https://www.orfonline.org/research/gender-wage-gap-in-the-last-ten-years-a-case-study-of-india/

[bibr23-0020715220985922] Hagqvist E Gådin KG Nordenmark M (2017) Work–family conflict and well-being across Europe: The role of gender context. Social Indicators Research 132: 785–797.

[bibr24-0020715220985922] Hamamura T (2011) Are cultures becoming individualistic? A cross-temporal comparison of individualism–collectivism in the United States and Japan. Personality and Social Psychology Review 16: 3–24. 21700795 10.1177/1088868311411587

[bibr25-0020715220985922] Hertog E (2009) Tough Choices: Bearing an Illegitimate Child in Japan. Stanford, CA: Stanford University Press.

[bibr26-0020715220985922] ISSP Research Group (2016) International social survey programme. ZA4850 Data File Version 2.0.0, ed. Cologne. Available at: https://www.gesis.org/en/issp/search-and-data-access

[bibr27-0020715220985922] Jalovaara M Neyer G Andersson G, et al. (2017) Education, gender, and Cohort fertility in the Nordic countries In: Department of Sociology SU (ed.) Stockholm Research Reports in Demography. Stockholm, pp. 27. Available at: file:///C:/Users/User/Downloads/Jalovaaraetal.2017SUDA.pdf

[bibr28-0020715220985922] Kalmijn M (2009) Country differences in the effects of divorce on well-being: The role of norms, support, and selectivity. European Sociological Review 26: 475–490.

[bibr29-0020715220985922] Kalmijn M (2011) The influence of men’s income and employment on marriage and cohabitation: Testing Oppenheimer’s Theory in Europe. European Journal of Population/Revue européenne de Démographie 27: 269–293. 21957323 10.1007/s10680-011-9238-x PMC3163818

[bibr30-0020715220985922] Kalmijn M (2013) The educational gradient in marriage: A comparison of 25 European countries. Demography 50: 1499–1520. 23821472 10.1007/s13524-013-0229-x

[bibr31-0020715220985922] Katsurada E Sugihara Y (2002) Gender-role identity, attitudes toward marriage, and gender-segregated school backgrounds. Sex Roles 47: 249–258.

[bibr32-0020715220985922] Lesthaeghe R (2010) The unfolding story of the second demographic transition. Population and Development Review 36: 211–251. 20734551 10.1111/j.1728-4457.2010.00328.x

[bibr33-0020715220985922] Lesthaeghe R Meekers D (1987) Value changes and the dimensions of familism in the European Community. European Journal of Population 2: 225–268.

[bibr34-0020715220985922] Lesthaeghe R Van De Kaa DJ (1986) Two demographic transitions. In: van de Kaa DJ Lesthaeghe R (eds) Population: Growth and Decline. Deventer: Van Loghum Slaterus, pp. 9–24.

[bibr35-0020715220985922] McDonald P (2000) Gender equity in theories of fertility transition. Population and Development Review 26: 427–439.

[bibr36-0020715220985922] Mensch BS Singh S Casterline JB (2005) Trends in the timing of first marriage among men and women in the developing world. In: Looyd CB Behrman JR Stromquist NP, et al. (eds) The Changing Transitions to Adulthood in Developing Countries: Selected Studies. Washington, DC: The National Academies Press, pp. 118–171.

[bibr37-0020715220985922] Nemoto K (2008) Postponed marriage: Exploring women’s views of matrimony and work in Japan. Gender & Society 22: 219–237.

[bibr38-0020715220985922] Ohlsson Wijk S Brandén M Duvander A-Z (2018) Committing to Marriage? The Role of Marriage Attitudes and Gender Equality among Young Cohabiters in Sweden. Stockholm: Stockholm Research Reports in Demography.

[bibr39-0020715220985922] Pessin L (2018) Changing gender norms and marriage dynamics in the United States. Journal of Marriage and Family 80: 25–41. 29335657 10.1111/jomf.12444 PMC5766036

[bibr40-0020715220985922] Rindfuss RR Choe MK Brauner-Otto SR (2016) The emergence of two distinct fertility regimes in economically advanced countries. Population Research and Policy Review 35: 287–304. 29593366 10.1007/s11113-016-9387-z PMC5868979

[bibr41-0020715220985922] Stanik CE McHale SM Crouter AC (2013) Gender dynamics predict changes in marital love among African American couples. Journal of Marriage and Family 75: 795–807. 23956462 10.1111/jomf.12037 PMC3744219

[bibr42-0020715220985922] Taniguchi H Kaufman G (2014) Gender role attitudes, troubles talk, and marital satisfaction in Japan. Journal of Social and Personal Relationships 31: 975–994.

[bibr43-0020715220985922] Treas J Lui J Gubernskaya Z (2014) Attitudes on marriage and new relationships: Cross-national evidence on the deinstitutionalization of marriage. Demographic Research 30: 1495–1526. 26052248 10.4054/DemRes.2014.30.54 PMC4455962

[bibr44-0020715220985922] United Nations Development Programme (2019) Human development reports—Gender Inequality Index (GII). Available at: http://hdr.undp.org/en/content/gender-inequality-index-gii

[bibr45-0020715220985922] United Nations (2019) World Fertility Data 2019. Available at: https://www.un.org/en/development/desa/population/publications/dataset/fertility/wfd2019.asp#:~:text=World%20Fertility%20Data%202019%20presents,or%20areas%20of%20the%20world

[bibr46-0020715220985922] Van De Kaa DJ (1987) Europe’s second demographic transition. Population Bulletin 42: 1–59. 12268395

[bibr47-0020715220985922] Van De Kaa DJ (2002) The Idea of a Second Demographic Transition in Industrialized Countries. Tokyo: National Institute of Population and Social Security Research.

[bibr48-0020715220985922] Van Der Meer T Te Grotenhuis M Pelzer B (2010) Influential cases in multilevel modeling: A methodological comment. American Sociological Review 75: 173–178.

[bibr49-0020715220985922] Wilcox WB Nock SL (2006) What’s love got to do with it? Equality, equity, commitment and women’s marital quality. Social Forces 84: 1321–1345.

[bibr50-0020715220985922] Yang W-S Yen P-C (2011) A comparative study of marital dissolution in East Asian societies: Gender attitudes and social expectations towards marriage in Taiwan, Korea and Japan. Asian Journal of Social Science 39: 751–775.

[bibr51-0020715220985922] Zhou M (2017) Motherhood, employment, and the dynamics of women’s gender attitudes. Gender & Society 31: 751–776.

[bibr52-0020715220985922] Zhou M Kan M-Y (2019) A new family equilibrium? Changing dynamics between the gender division of labor and fertility in Great Britain, 1991–2017. Demographic Research 40: 1455–1500.

