# Supplementary material for: Country differences in the link between gender-role attitudes and marital centrality: Evidence from 24 countries
Source: Int J Comp Sociol. 2021 Jan 7;61(5):291–309. doi: 10.1177/0020715220985922 (PMC7961650; doi:10.1177/0020715220985922)
Supplement: sj-pdf-1-cos-10.1177_0020715220985922.pdf – Supplemental material for Country differences in the link between gender-role attitudes and marital centrality: Evidence from 24 countries [file sj-pdf-1-cos-10.1177_0020715220985922.pdf.pdf]

Online Appendix

Table A1. Number of countries and number of observations in each country

Country	Name	Region/Welfare type	2002	2012	Total
AT	Australia	Conservative	1,013	818	1,831
AU	Austria	Liberal	831	807	1,638
BE	Belgium	Conservative	727	1,296	2,023
CH	Switzerland	Conservative	462	838	1,300
CZ	Czechia	Eastern European	786	1,122	1,908
DE	Germany	Conservative	692	1,087	1,779
DK	Denmark	Socio-democratic	708	977	1,685
ES	Spain	Mediterranean	1,363	1,632	2,995
FI	Finland	Socio-democratic	617	709	1,326
FR	France	Conservative	1,109	1,259	2,368
GB	United Kingdom	Liberal	1,186	502	1,688
HU	Hungry	Conservative	588	659	1,247
JP	Japan	East Asian	542	625	1,167
LV	Latvia	Eastern European	629	694	1,323
NL	Netherlands	Conservative	747	678	1,425
NO	Norway	Socio-democratic	789	885	1,674
PL	Poland	Eastern European	665	718	1,383
PT	Portugal	Mediterranean	653	622	1,275
RU	Russian Federation	Eastern European	950	881	1,831
SE	Sweden	Socio-democratic	556	588	1,144
SI	Slovenia	Eastern European	244	625	869
SK	Slovakia	Eastern European	744	683	1,427
TW	Taiwan	East Asian	731	1,548	2,279
US	United States	Liberal	777	891	1,668
Total			18,109	21,144	39,253

Table A2a. Women: Mean (SD) of key individual-level variables (not standardized) by country

	2002: Marriage Attitudes	2002: EGA	2012: Marriage Attitudes	2012: EGA
AT	7.402	19.765	7.149	19.922
	(2.787)	(5.079)	(2.882)	(5.302)
AU	8.754	20.139	7.518	21.344
	(2.540)	(4.682)	(2.575)	(4.340)
BE	6.088	19.725	6.609	21.644
	(2.169)	(4.523)	(2.677)	(4.773)
CH	6.979	19.748	7.476	20.188
	(2.210)	(4.202)	(2.447)	(4.472)
CZ	9.016	18.234	9.443	19.101
	(2.429)	(4.681)	(2.589)	(4.820)
DE	7.460	22.576	7.049	23.402
	(2.610)	(5.119)	(2.680)	(5.005)
DK	5.986	23.618	5.815	24.883
	(2.514)	(4.848)	(2.568)	(4.258)
ES	6.745	20.248	5.919	21.020
	(2.227)	(4.312)	(2.310)	(4.299)
FI	7.307	20.776	6.783	23.229
	(2.715)	(4.468)	(2.592)	(4.229)
FR	6.371	21.212	5.778	22.641
	(2.907)	(5.165)	(2.644)	(4.691)
GB	7.608	20.814	7.303	20.971
	(2.350)	(4.302)	(2.269)	(4.287)
HU	8.021	16.549	7.654	17.254
	(2.571)	(4.454)	(2.762)	(4.888)
JP	9.074	19.764	8.646	20.366
	(3.007)	(4.132)	(2.858)	(3.994)
LV	9.290	17.354	9.160	16.617
	(2.276)	(3.879)	(2.814)	(4.623)
NL	6.584	20.320	6.616	22.401
	(2.068)	(4.475)	(2.432)	(4.587)
NO	6.967	22.381	6.581	23.761
	(2.410)	(4.187)	(2.348)	(4.147)
PL	9.000	18.235	8.911	19.262
	(2.304)	(4.447)	(2.606)	(4.495)
PT	7.029	18.188	6.608	20.240
	(2.551)	(4.395)	(2.371)	(3.966)
RU	9.420	16.480	10.393	16.888
	(2.387)	(4.051)	(2.427)	(4.217)
SE	6.238	23.156	6.060	24.495
	(2.379)	(4.341)	(2.348)	(3.873)
SI	7.593	19.089	6.534	21.701
	(2.481)	(4.285)	(2.401)	(4.402)
SK	10.484	17.465	9.862	18.530
	(2.776)	(4.816)	(2.977)	(4.340)

TW	9.552	18.212	8.926	18.581
	(1.950)	(2.612)	(2.200)	(3.072)
US	9.099	20.693	8.834	20.130
	(2.953)	(5.569)	(2.446)	(3.965)
Total	7.796	19.832	7.512	20.817
	(2.808)	(4.912)	(2.907)	(4.925)
N	10453	10453	11635	11635

Table A2b. Men: Mean (SD) of key individual-level variables (not standardized) by country

	2002: Marriage Attitudes	2002: EGA	2012: Marriage Attitudes	2012: EGA
AT	8.036	18.184	7.668	18.448
	(2.692)	(4.906)	(3.015)	(4.759)
AU	9.298	18.573	8.264	19.826
	(2.485)	(4.464)	(2.682)	(4.526)
BE	6.672	18.704	7.169	20.621
	(2.291)	(4.492)	(2.837)	(4.435)
CH	7.727	18.732	7.743	19.120
	(2.177)	(4.538)	(2.274)	(4.060)
CZ	8.942	17.670	9.714	18.200
	(2.565)	(4.004)	(2.613)	(4.663)
DE	7.988	20.932	7.558	21.875
	(2.547)	(4.560)	(2.517)	(4.860)
DK	6.176	22.962	6.256	23.966
	(2.621)	(4.973)	(2.646)	(4.454)
ES	6.955	19.275	6.500	20.229
	(2.199)	(4.180)	(2.504)	(4.414)
FI	8.094	19.376	7.409	21.571
	(2.742)	(4.360)	(2.496)	(4.290)
FR	6.845	20.052	6.374	22.042
	(2.809)	(5.275)	(2.869)	(4.749)
GB	8.119	19.380	7.960	20.280
	(2.235)	(4.088)	(2.357)	(4.014)
HU	8.181	16.338	7.843	17.000
	(2.399)	(4.195)	(2.448)	(4.483)
JP	8.961	19.335	9.313	20.320
	(2.944)	(4.295)	(2.489)	(3.946)
LV	9.107	17.190	8.903	16.930
	(2.585)	(4.003)	(2.461)	(3.914)
NL	7.178	19.648	6.710	21.357
	(2.232)	(4.391)	(2.284)	(4.544)
NO	7.524	20.928	7.243	21.981
	(2.418)	(4.351)	(2.403)	(4.198)
PL	9.627	17.199	8.997	18.283
	(1.973)	(4.185)	(2.261)	(3.797)
PT	7.576	17.361	7.074	19.488
	(2.499)	(4.182)	(2.373)	(4.075)
RU	9.390	16.246	10.129	16.430
	(2.291)	(3.884)	(2.142)	(3.785)
SE	6.888	20.996	6.863	22.765
	(2.387)	(4.809)	(2.655)	(4.306)
SI	8.844	18.046	7.134	20.600
	(2.590)	(3.999)	(2.476)	(3.998)
SK	10.237	16.616	10.417	17.879
	(2.781)	(4.265)	(2.624)	(4.309)
TW	9.763	17.564	9.258	17.945
	(2.054)	(2.614)	(2.166)	(2.942)

US	9.958	19.000	9.183	18.942
	(2.564)	(5.077)	(2.303)	(3.669)
Total	8.203	18.813	7.948	19.850
	(2.699)	(4.628)	(2.783)	(4.634)
N	7656	7656	9509	9509

Table A3a. Mean of control variables in 2002

	Single	Married	Sep/Div/Wid	No child	1 child	2 children	>2 children
AT	0.29	0.54	0.17	0.54	0.28	0.15	0.03
AU	0.15	0.73	0.12	0.68	0.12	0.14	0.06
BE	0.25	0.64	0.11	0.57	0.22	0.16	0.05
CH	0.31	0.52	0.17	0.59	0.22	0.14	0.05
CZ	0.22	0.62	0.17	0.54	0.29	0.15	0.02
DE	0.28	0.61	0.11	0.58	0.26	0.13	0.03
DK	0.36	0.54	0.11	0.52	0.25	0.19	0.05
ES	0.36	0.55	0.09	0.59	0.28	0.12	0.01
FI	0.12	0.80	0.08	0.55	0.23	0.17	0.04
FR	0.26	0.61	0.14	0.46	0.30	0.19	0.05
GB	0.24	0.61	0.15	0.58	0.22	0.16	0.04
HU	0.20	0.60	0.20	0.55	0.28	0.14	0.04
JP	0.19	0.77	0.04	0.52	0.20	0.23	0.06
LV	0.21	0.61	0.17	0.44	0.35	0.17	0.04
NL	0.32	0.59	0.09	0.60	0.17	0.17	0.06
NO	0.30	0.56	0.14	0.49	0.24	0.22	0.06
PL	0.21	0.67	0.12	0.42	0.32	0.19	0.07
PT	0.28	0.62	0.11	0.54	0.33	0.11	0.02
RU	0.16	0.59	0.25	0.43	0.41	0.15	0.01
SE	0.20	0.75	0.05	0.50	0.21	0.21	0.07
SI	0.19	0.77	0.04	0.00	0.64	0.30	0.07
SK	0.22	0.67	0.11	0.51	0.28	0.17	0.04
TW	0.34	0.61	0.05	0.48	0.24	0.19	0.09
US	0.27	0.50	0.23	0.61	0.21	0.11	0.06
Total	0.25	0.62	0.13	0.53	0.27	0.16	0.04
N	18109	18109	18109	18109	18109	18109	18109

	Full-time working	Part-time working	Not working	<Sec. education	Secondary	>Sec. edu	Has religion	Age
AT	0.67	0.00	0.33	0.68	0.12	0.20	0.85	39.78
AU	0.47	0.21	0.32	0.46	0.16	0.38	0.72	43.02
BE	0.59	0.19	0.22	0.27	0.36	0.37	0.77	40.34
CH	0.58	0.27	0.15	0.54	0.11	0.35	0.82	41.63
CZ	0.71	0.03	0.26	0.52	0.37	0.11	0.35	39.03
DE	0.59	0.10	0.31	0.66	0.12	0.22	0.66	39.72
DK	0.72	0.09	0.19	0.09	0.42	0.49	0.92	40.10
ES	0.56	0.12	0.33	0.55	0.24	0.21	0.79	38.01
FI	0.68	0.09	0.23	0.15	0.60	0.25	0.90	41.43
FR	0.62	0.16	0.22	0.36	0.13	0.51	0.63	38.74
GB	0.61	0.17	0.22	0.46	0.17	0.38	0.51	40.07
HU	0.59	0.04	0.38	0.47	0.35	0.18	0.93	41.09
JP	0.54	0.17	0.29	0.11	0.49	0.40	0.30	42.02
LV	0.73	0.08	0.20	0.12	0.21	0.67	0.65	37.86
NL	0.44	0.32	0.24	0.18	0.42	0.40	0.33	41.77
NO	0.67	0.10	0.22	0.22	0.35	0.42	1.00	40.73
PL	0.52	0.07	0.41	0.46	0.29	0.26	0.90	40.04
PT	0.65	0.06	0.29	0.61	0.14	0.25	0.89	38.61
RU	0.68	0.09	0.23	0.07	0.30	0.63	0.74	38.98
SE	0.66	0.17	0.17	0.41	0.21	0.38	0.79	40.95
SI	0.77	0.01	0.21	0.47	0.34	0.19	0.83	38.08
SK	0.70	0.04	0.26	0.49	0.40	0.10	0.88	38.81
TW	0.66	0.06	0.28	0.29	0.30	0.41	0.73	36.59
US	0.63	0.12	0.24	0.11	0.30	0.59	0.84	38.38
Total	0.62	0.12	0.26	0.37	0.28	0.35	0.73	39.73
N	18109	18109	18109	18109	18109	18109	18109	18109

Table A3b. Mean of control variables in 2012

	Single	Married	Sep/Div/Wid	No child	1 child	2 children	>2 children
AT	0.23	0.63	0.14	0.69	0.15	0.11	0.05
AU	0.31	0.60	0.10	0.56	0.16	0.19	0.09
BE	0.28	0.59	0.13	0.56	0.18	0.19	0.07
CH	0.35	0.56	0.09	0.60	0.18	0.16	0.07
CZ	0.23	0.59	0.18	0.60	0.20	0.17	0.02
DE	0.35	0.52	0.13	0.63	0.20	0.14	0.04
DK	0.39	0.49	0.12	0.56	0.09	0.18	0.16
ES	0.31	0.61	0.08	0.55	0.24	0.17	0.04
FI	0.38	0.52	0.10	0.56	0.18	0.18	0.07
FR	0.27	0.59	0.14	0.43	0.25	0.24	0.08
GB	0.37	0.42	0.21	0.51	0.21	0.22	0.06
HU	0.34	0.42	0.24	0.61	0.19	0.13	0.07
JP	0.26	0.68	0.06	0.54	0.18	0.19	0.09
LV	0.26	0.52	0.22	0.57	0.26	0.15	0.02
NL	0.30	0.60	0.10	0.57	0.18	0.17	0.08
NO	0.37	0.53	0.11	0.51	0.20	0.20	0.10
PL	0.27	0.63	0.10	0.49	0.24	0.18	0.09
PT	0.37	0.49	0.14	0.61	0.23	0.13	0.03
RU	0.26	0.51	0.22	0.59	0.26	0.13	0.02
SE	0.40	0.49	0.11	0.54	0.21	0.19	0.06
SI	0.25	0.69	0.07	0.59	0.22	0.15	0.04
SK	0.18	0.69	0.13	0.59	0.19	0.16	0.05
TW	0.35	0.58	0.07	0.52	0.21	0.20	0.07
US	0.34	0.46	0.20	0.60	0.16	0.15	0.08
Total	0.31	0.56	0.13	0.56	0.20	0.17	0.06
N	21144	21144	21144	21144	21144	21144	21144

	Full-time working	Part-time working	Not working	<Sec. education	Secondary	>Sec. edu	Has religion	Age
AT	0.62	0.20	0.19	0.65	0.09	0.26	0.83	39.99
AU	0.52	0.31	0.16	0.16	0.18	0.66	0.59	42.77
BE	0.51	0.23	0.26	0.20	0.26	0.54	0.63	40.70
CH	0.58	0.26	0.17	0.15	0.05	0.80	0.74	40.87
CZ	0.75	0.04	0.20	0.30	0.57	0.13	0.23	40.91
DE	0.55	0.24	0.21	0.08	0.04	0.88	0.64	41.01
DK	0.60	0.18	0.21	0.08	0.07	0.85	0.83	40.11
ES	0.49	0.17	0.33	0.39	0.15	0.46	0.74	40.35
FI	0.66	0.15	0.19	0.06	0.34	0.60	0.75	41.34
FR	0.43	0.38	0.19	0.31	0.18	0.51	0.50	40.94
GB	0.42	0.29	0.29	0.38	0.22	0.40	0.44	40.64
HU	0.60	0.05	0.35	0.46	0.31	0.23	0.78	40.62
JP	0.58	0.24	0.18	0.06	0.41	0.53	0.28	41.25
LV	0.64	0.08	0.27	0.11	0.29	0.60	0.66	40.33
NL	0.36	0.43	0.21	0.14	0.13	0.73	0.54	43.41
NO	0.73	0.17	0.10	0.17	0.21	0.62	0.78	40.88
PL	0.63	0.10	0.27	0.10	0.59	0.31	0.84	39.82
PT	0.61	0.12	0.27	0.47	0.31	0.22	0.83	39.97
RU	0.63	0.09	0.29	0.05	0.14	0.81	0.86	39.54
SE	0.67	0.18	0.15	0.23	0.25	0.51	0.77	41.66
SI	0.67	0.04	0.29	0.32	0.36	0.32	0.68	40.53
SK	0.69	0.05	0.25	0.37	0.43	0.19	0.85	43.81
TW	0.64	0.12	0.24	0.20	0.29	0.51	0.80	39.42
US	0.51	0.18	0.30	0.12	0.52	0.36	0.76	39.48
Total	0.58	0.18	0.24	0.23	0.26	0.51	0.68	40.74
N	21144	21144	21144	21144	21144	21144	21144	21144

Table A4. Key variables of interest by country

Country	Year	Sex	Marital centrality (by sex)	Std. Marital centrality (by sex)	EGA (by sex)	Std. EGA (by sex)	Country-level: Gender Inequality Index (GII)	Country-level: Total Fertility Rate
AT	2002	Women	7.40	-0.40	19.76	-0.01	0.15	1.77
AT	2002	Men	8.04	0.10	18.18	-0.77	0.15	1.77
AT	2012	Women	7.15	-0.60	19.92	0.06	0.10	1.93
AT	2012	Men	7.67	-0.19	18.45	-0.64	0.10	1.93
AU	2002	Women	8.75	0.67	20.14	0.17	0.16	1.77
AU	2002	Men	9.30	1.10	18.57	-0.58	0.16	1.77
AU	2012	Women	7.52	-0.31	21.34	0.75	0.13	1.93
AU	2012	Men	8.26	0.28	19.83	0.02	0.13	1.93
BE	2002	Women	6.09	-1.44	19.73	-0.03	0.12	1.65
BE	2002	Men	6.67	-0.98	18.70	-0.52	0.12	1.65
BE	2012	Men	7.17	-0.59	20.62	0.40	0.08	1.8
BE	2012	Women	6.61	-1.03	21.64	0.89	0.08	1.8
CH	2002	Men	7.73	-0.15	18.73	-0.51	0.09	1.39
CH	2002	Women	6.98	-0.74	19.75	-0.02	0.09	1.39
CH	2012	Men	7.74	-0.13	19.12	-0.32	0.06	1.53
CH	2012	Women	7.48	-0.34	20.19	0.19	0.06	1.53
CZ	2002	Men	8.94	0.82	17.67	-1.02	0.15	1.18
CZ	2002	Women	9.02	0.87	18.23	-0.74	0.15	1.18
CZ	2012	Women	9.44	1.21	19.10	-0.33	0.13	1.46
CZ	2012	Men	9.71	1.43	18.20	-0.76	0.13	1.46
DE	2002	Men	7.99	0.06	20.93	0.55	0.13	1.33
DE	2002	Women	7.46	-0.36	22.58	1.34	0.13	1.33
DE	2012	Men	7.56	-0.28	21.88	1.00	0.09	1.41

Country	Year	Sex	Marital centrality (by sex)	Std. Marital centrality (by sex)	EGA (by sex)	Std. EGA (by sex)	Country-level: Gender Inequality Index (GII)	Country-level: Total Fertility Rate
DE	2012	Women	7.05	-0.68	23.40	1.73	0.09	1.41
DK	2002	Men	6.18	-1.37	22.96	1.52	0.08	1.73
DK	2002	Women	5.99	-1.52	23.62	1.84	0.08	1.73
DK	2012	Women	5.82	-1.66	24.88	2.44	0.05	1.72
DK	2012	Men	6.26	-1.31	23.97	2.00	0.05	1.72
ES	2002	Men	6.96	-0.76	19.27	-0.25	0.12	1.26
ES	2002	Women	6.75	-0.92	20.25	0.22	0.12	1.26
ES	2012	Women	5.92	-1.58	21.02	0.59	0.10	1.33
ES	2012	Men	6.50	-1.12	20.23	0.21	0.10	1.33
FI	2002	Women	7.31	-0.48	20.78	0.47	0.09	1.71
FI	2002	Men	8.09	0.14	19.38	-0.20	0.09	1.71
FI	2012	Women	6.78	-0.89	23.23	1.65	0.07	1.81
FI	2012	Men	7.41	-0.40	21.57	0.85	0.07	1.81
FR	2002	Women	6.37	-1.22	21.21	0.68	0.17	1.86
FR	2002	Men	6.84	-0.84	20.05	0.13	0.17	1.86
FR	2012	Women	5.78	-1.69	22.64	1.37	0.09	2
FR	2012	Men	6.37	-1.22	22.04	1.08	0.09	2
GB	2002	Men	8.12	0.16	19.38	-0.20	0.22	1.65
GB	2002	Women	7.61	-0.24	20.81	0.49	0.22	1.65
GB	2012	Men	7.96	0.04	20.28	0.24	0.17	1.92
GB	2012	Women	7.30	-0.48	20.97	0.57	0.17	1.92
HU	2002	Women	8.02	0.09	16.55	-1.55	0.29	1.31
HU	2002	Men	8.18	0.21	16.34	-1.65	0.29	1.31
HU	2012	Women	7.65	-0.20	17.25	-1.21	0.26	1.34
HU	2012	Men	7.84	-0.05	17.00	-1.34	0.26	1.34
JP	2002	Women	9.07	0.92	19.76	-0.01	0.14	1.32

Country	Year	Sex	Marital centrality (by sex)	Std. Marital centrality (by sex)	EGA (by sex)	Std. EGA (by sex)	Country-level: Gender Inequality Index (GII)	Country-level: Total Fertility Rate
JP	2002	Men	8.96	0.83	19.33	-0.22	0.14	1.32
JP	2012	Women	8.65	0.58	20.37	0.28	0.13	1.41
JP	2012	Men	9.31	1.11	20.32	0.25	0.13	1.41
LV	2002	Women	9.29	1.09	17.35	-1.17	0.28	1.25
LV	2002	Men	9.11	0.95	17.19	-1.25	0.28	1.25
LV	2012	Men	8.90	0.79	16.93	-1.37	0.19	1.45
LV	2012	Women	9.16	0.99	16.62	-1.52	0.19	1.45
NL	2002	Men	7.18	-0.58	19.65	-0.07	0.10	1.74
NL	2002	Women	6.58	-1.05	20.32	0.25	0.10	1.74
NL	2012	Women	6.62	-1.02	22.40	1.25	0.05	1.71
NL	2012	Men	6.71	-0.95	21.36	0.75	0.05	1.71
NO	2002	Women	6.97	-0.75	22.38	1.24	0.11	1.76
NO	2002	Men	7.52	-0.31	20.93	0.55	0.11	1.76
NO	2012	Women	6.58	-1.05	23.76	1.90	0.07	1.85
NO	2012	Men	7.24	-0.53	21.98	1.05	0.07	1.85
PL	2002	Men	9.63	1.36	17.20	-1.24	0.21	1.25
PL	2002	Women	9.00	0.86	18.24	-0.74	0.21	1.25
PL	2012	Women	8.91	0.79	19.26	-0.25	0.15	1.34
PL	2012	Men	9.00	0.86	18.28	-0.72	0.15	1.34
PT	2002	Women	7.03	-0.70	18.19	-0.77	0.21	1.47
PT	2002	Men	7.58	-0.26	17.36	-1.16	0.21	1.47
PT	2012	Women	6.61	-1.03	20.24	0.22	0.13	1.28
PT	2012	Men	7.07	-0.66	19.49	-0.14	0.13	1.28
RU	2002	Women	9.42	1.19	16.48	-1.59	0.42	1.29
RU	2002	Men	9.39	1.17	16.25	-1.70	0.42	1.29
RU	2012	Women	10.39	1.96	16.89	-1.39	0.30	1.71

Country	Year	Sex	Marital centrality (by sex)	Std. Marital centrality (by sex)	EGA (by sex)	Std. EGA (by sex)	Country-level: Gender Inequality Index (GII)	Country-level: Total Fertility Rate
RU	2012	Men	10.13	1.76	16.43	-1.61	0.30	1.71
SE	2002	Men	6.89	-0.81	21.00	0.58	0.06	1.65
SE	2002	Women	6.24	-1.32	23.16	1.61	0.06	1.65
SE	2012	Men	6.86	-0.83	22.76	1.43	0.05	1.9
SE	2012	Women	6.06	-1.46	24.50	2.26	0.05	1.9
SI	2002	Men	8.84	0.74	18.05	-0.84	0.14	1.21
SI	2002	Women	7.59	-0.25	19.09	-0.34	0.14	1.21
SI	2012	Men	7.13	-0.61	20.60	0.39	0.07	1.59
SI	2012	Women	6.53	-1.09	21.70	0.92	0.07	1.59
SK	2002	Men	10.24	1.84	16.62	-1.52	0.22	1.19
SK	2002	Women	10.48	2.04	17.46	-1.11	0.22	1.19
SK	2012	Women	9.86	1.54	18.53	-0.60	0.19	1.34
SK	2012	Men	10.42	1.98	17.88	-0.92	0.19	1.34
TW	2002	Women	9.55	1.30	18.21	-0.76	0.22	1.34
TW	2002	Men	9.76	1.46	17.56	-1.07	0.22	1.34
TW	2012	Men	9.26	1.07	17.95	-0.88	0.05	1.27
TW	2012	Women	8.93	0.80	18.58	-0.58	0.05	1.27
US	2002	Women	9.10	0.94	20.69	0.43	0.26	2.02
US	2002	Men	9.96	1.62	19.00	-0.38	0.26	2.02
US	2012	Men	9.18	1.01	18.94	-0.41	0.24	1.88
US	2012	Women	8.83	0.73	20.13	0.16	0.24	1.88
